# Serum plasminogen activator inhibitor 1 (PAI-1) and intra-ocular pressure (IOP): the Guangzhou biobank cohort study

**DOI:** 10.1186/1753-6561-6-S4-O26

**Published:** 2012-07-09

**Authors:** J Heggie, K Tanner, M Protty, GN Thomas

**Affiliations:** 1 College of Medical and Dental Sciences, University of Birmingham, UK

## Introduction

Increased intraocular pressure (IOP) is a major risk factor for developing ocular disease such as primary open-angle glaucoma. Plasminogen activator inhibitor 1 (PAI-1) plays a role in the turnover and degradation of extracellular matrix proteins (ECM) which are thought to play a role in the pathogenesis of increased IOP. To determine if an independent association exists between serum PAI-1 and IOP in humans.

## Methods

A cross-sectional study with participants from the Guangzhou biobank cohort study (GBCS-CVS) aged 50–85 years were recruited and received a medical check-up including measurement of serum PAI-1, IOP, blood pressure, fasting LDL- and HDL-cholesterol, glucose and obesity measures. Information on socioeconomic and lifestyle factors was also collected. Subjects were divided into tertiles based on serum PAI-1 and a logistic regression analysis was performed to derive an odds ratio for having high IOP for each tertile. Personal, social and vascular confounders were adjusted for.

## Results

The risk of increased IOP was significantly raised with higher serum PAI-1, with adjusted odds ratios (95% confidence intervals) for second and third tertiles of 1.31 (0.81-2.12) and 1.81 (1.15-2.83), respectively. Haematocrit, glycosylated haemoglobin (HbA1c), heart rate, and high-density lipoprotein (HDL-C) were the only vascular risk factors positively associated with serum PAI-1 levels (p from 0.03 to <0.01).

## Conclusions

There is a strong relationship between serum PAI-1 and IOP in this older Chinese population. Further studies are needed to confirm these findings in this and other populations.

